# Dance Intervention Impact on Brain Plasticity: A Randomized 6-Month fMRI Study in Non-expert Older Adults

**DOI:** 10.3389/fnagi.2021.724064

**Published:** 2021-10-27

**Authors:** Zuzana Balazova, Radek Marecek, L’ubomíra Novakova, Nela Nemcova-Elfmarkova, Sylvie Kropacova, Luboš Brabenec, Roman Grmela, Pavlína Vaculíková, Lenka Svobodova, Irena Rektorova

**Affiliations:** ^1^Brain and Mind Research, Central European Institute of Technology, Masaryk University, Brno, Czechia; ^2^Faculty of Medicine, Masaryk University, Brno, Czechia; ^3^First Department of Neurology, Faculty of Medicine, St. Anne’s University Hospital, Masaryk University, Brno, Czechia; ^4^Department of Health Promotion, Faculty of Sports Studies, Masaryk University, Brno, Czechia; ^5^Department of Gymnastics and Combatives, Faculty of Sports Studies, Masaryk University, Brno, Czechia

**Keywords:** dance intervention, resting state fMRI, independent component analysis, intra-network connectivity, attention, cognitive

## Abstract

**Background:** Dance is a complex activity combining physical exercise with cognitive, social, and artistic stimulation.

**Objectives:** We aimed to assess the effects of dance intervention (DI) on intra and inter-network resting-state functional connectivity (rs-FC) and its association to cognitive changes in a group of non-demented elderly participants.

**Methods:** Participants were randomly assigned into two groups: DI and life as usual (LAU). Six-month-long DI consisted of supervised 60 min lessons three times per week. Resting-state fMRI data were processed using independent component analysis to evaluate the intra and inter-network connectivity of large-scale brain networks. Interaction between group (DI, LAU) and visit (baseline, follow-up) was assessed using ANOVA, and DI-induced changes in rs-FC were correlated with cognitive outcomes.

**Results:** Data were analyzed in 68 participants (DI; *n* = 36 and LAU; *n* = 32). A significant behavioral effect was found in the attention domain, with Z scores increasing in the DI group and decreasing in the LAU group (*p* = 0.017). The DI as compared to LAU led to a significant rs-FC increase of the default mode network (DMN) and specific inter-network pairings, including insulo-opercular and right frontoparietal/frontoparietal control networks (*p* = 0.019 and *p* = 0.023), visual and language/DMN networks (*p* = 0.012 and *p* = 0.015), and cerebellar and visual/language networks (*p* = 0.015 and *p* = 0.003). The crosstalk of the insulo-opercular and right frontoparietal networks were associated with attention/executive domain Z-scores (*R* = 0.401, *p* = 0.015, and *R* = 0.412, *p* = 0.012).

**Conclusion:** The DI led to intervention-specific complex brain plasticity changes that were of cognitive relevance.

## Introduction

Dance is a complex activity combining physical exercise with cognitive, social, and artistic stimulation (Burzynska et al., 2017). While the beneficial effect of dance intervention (DI) programs on physical fitness have been repeatedly proven, papers assessing the effects of DI on cognition have mixed results (Alpert et al., 2009; Coubard, 2011; Kim et al., 2011; Kattenstroth et al., 2013; Müller et al., 2017; Rehfeld et al., 2017, 2018; Brustio et al., 2018; Qi et al., 2018b). The effects of dance intervention on brain plasticity include various structural changes, such as an increase in gray matter volume (left hippocampus, left dentate gyrus, left precentral gyrus, etc.) as well as an increase in white matter integrity (fornix, corpus callosum), for a full review please see Teixeira-Machado et al. (2019). These findings are in concordance with our recent works, where 6-month-long DI as compared to life activities as usual (LAU) and resulted in significant improvement in a Five Point Test (FPT) which evaluated attention and executive functions (Kropacova et al., 2019), and in increases of cortical thickness of the right lateral occipitotemporal cortex (Rektorova et al., 2020) implicated in learning new skilled movements (Gatti et al., 2017). Physical fitness also significantly improved due to the 6-month DI, which was linked to changes in diffusion tensor imagining measures in the whole white matter skeleton and in the corticospinal tract and the superior longitudinal fascicle, which are engaged in motor learning and movement execution, as well as in spatial attention, manipulation of mental representations, and speech comprehension (Sejnoha Minsterova et al., 2020).

Brain connectivity changes following aerobic exercise of various types and intensities were studied by several authors. Increase of resting-state functional connectivity (rsFC) following aerobic exercise was often reported in the default mode network (DMN) and hippocampus and parahippocampal gyrus regions (Burdette et al., 2010; Rosano et al., 2010; Voss et al., 2010; Leavitt et al., 2014; Boraxbekk et al., 2016; Tozzi et al., 2016; Chirles et al., 2017; McGregor et al., 2018; Ikuta et al., 2019). A 6-month-long aerobic exercise intervention in MCI patients led to increased rsFC of the prefrontal cortex (Hugenschmidt et al., 2017), similarly, the aerobic exercise intervention of the same duration led to increased rsFC in the prefrontal cortex, as well as superior parietal gyrus/precuneus in older overweight adults compared to the control group (Prehn et al., 2019). Not only regular but also a single session of moderate aerobic exercise resulted in increased FC in the sensorimotor areas (pre/post central gyri, thalamus and secondary somatosensory area) of healthy young adults (Rajab et al., 2014). Similarly, a 30-min long exercise bout was reported to increase rsFC in the right fronto parietal network (rFPN), sensorimotor network (SMN), and right affect and reward and network (ARN) in male athletes (Schmitt et al., 2019). However, some studies reported limited or no effect of aerobic exercise on rsFC (Flodin et al., 2017; Cui et al., 2019).

Although dance, unlike simple aerobic activity, involves learning and attention, emotions, coordination and balance, acoustic stimulation and social interaction (Kshtriya et al., 2015) only one study to date (Qi et al., 2018b) used rs-fMRI to examine the effect of the intervention on FC. The results of this pilot 3-month-long aerobic dance intervention in older adults with MCI showed an increase in brain spontaneous activity (analysis of the amplitude of low-frequency fluctuations) in bilateral fronto temporal, hippocampal, entorhinal and anterior cingulate cortices, compared to baseline (Qi et al., 2018b).

Based on this promising preliminary finding, we decided to use fMRI and a data-driven independent componential analysis (ICA) approaches with a primary objective to assess the effect of DI on rsFC in a mixed group of healthy elderly with or without MCI but no dementia. We hypothesized that DI-induced task-specific changes in both within-network and between-network rsFC in the DI group compared to the LAU group. Our secondary objective was to evaluate the association between the DI-induced changes in rsFC and cognitive performance. Based on our previous results (Kropacova et al., 2019; Rektorova et al., 2020), we particularly focused on DI-induced changes in attention and executive function domains. We hypothesized that DI-induced brain plasticity changes will be linked with our cognitive outcomes of interest.

## Methods

### Study Participants and Intervention

Volunteers older than 60 were recruited using several sources, such as free advertising in community centers (senior centers, libraries), University of the Third Age courses, and in public media such as local newspapers and leaflets, etc. The exclusion criteria included serious brain injury, major psychiatric disorder or central nervous system disease, other serious neurological, orthopedic, oncological or internal disease disabling participation in the dance-exercise intervention, as well as any contraindications of MRI scans as followed in clinical practice. The abuse of alcohol, other addictive substances and smoking were other exclusion criteria. Volunteers practizing sport activities more frequently or at a more demanding level than the dance exercise intervention were not included in the study.

Participants were randomly assigned into two groups in a 1:1 ratio: DI (dance intervention) and LAU (life as usual) using the opaque envelope method. The study sample was estimated based on pilot data analysis, when a mean change of 0.5 points was observed in the five-point test score (Kropacova et al., 2019). Considering the type 1 error probability α of 0.05 and expecting the test power β of 0.8, and also considering a dropout rate of 25%, the minimum number of participants in one group was calculated to be 42. It should be noted that in the presented paper, the five point test is not included in the analysis. Participants who were eventually included in the analysis were those who completed the study protocol (at least 66.6% attendance of dance-exercise intervention in DI group, 2 fMRI scans, 2 detailed neuropsychological examinations before and after the intervention and physical fitness assessment).

Participants included in the DI group followed a 6-month-long dance exercise course taking place three times a week, the duration of a single lesson was 60 min and it took place at the Faculty of Sports Studies, Masaryk University in Brno. The dance exercise training was prepared and led by professionals from the faculty. The intervention included various dance types, e.g., Irish country, African, and Greek, etc., which were combined into a final choreography and performed at medium physical load intensity three times per week (Kropacova et al., 2019; Rektorova et al., 2020). Participants in the LAU group led normal lives.

### Neuropsychological Examination

The complex neuropsychological testing evaluated global cognitive functions (Montreal Cognitive Assessment—MOCA) and five domains: memory, attention, executive functions, visuospatial functions, and language. Subjects who scored below –1.5 SD in two tests in at least one cognitive domain compared to normative data were categorized as having MCI. Activities of daily living and depressive symptoms were also evaluated to exclude subjects with dementia or depression (Kropacova et al., 2019). For further details, see the Supplementary Material.

### MRI Data Acquisition and Preprocessing

All subjects were scanned in 3T Siemens Prisma MR scanner (Siemens Corp., Erlangen, Germany) at the Central European Institute of Technology (CEITEC), Masaryk University in Brno, using the following sequences: magnetization-prepared rapid gradient-echo (MPRAGE) high-resolution T1-weighted images (240 sagittal slices, slice thickness = 1 mm, TR = 2,300 ms, TE = 2.34 ms, FA = 8°, FOV = 224 mm, matrix size 224 × 224) and gradient-echo echo-planar imaging sequence for resting state fMRI (200 images, 34 transversal slices, slice thickness = 3.5 mm, TR = 1,990 ms, TE = 35 ms, FA = 70°, FOV = 192 mm, matrix size 64 × 64).

The resting state fMRI data were preprocessed using the SPM 12 toolbox and Matlab 2014b. Preprocessing included realignment and unwarping, normalization into standard anatomical space (MNI) and spatial smoothing with 5 mm FWHM. The level of motion was thoroughly checked in terms of frame-wise displacement (FD) (Power et al., 2012). No FD was higher than 3 mm and scans that displayed FD > 0.75 mm were scrubbed (Power et al., 2012). No more than 2.5% of subject scans were removed. Moreover, the six movement regressors (obtained during realignment and unwarping), FD and extracted signals from white matter and cerebrospinal fluid were regressed out of the data in subsequent analysis. Assessment of functional connectivity changes using independent component analysis (ICA).

Regarding the rs-fMRI data analyses, we utilized the data-driven approach using independent component analysis (ICA) methods. The intra-network and inter-network connectivity (intra-NC and inter-NC) of major large-scale brain networks were also evaluated, see the details below.

The data from all subjects (*n* = 68, 36 DI and 32 LAU) and both sessions (a total of 136 fMRI datasets) were entered into single group spatial Independent Component Analysis (gsICA) (Calhoun et al., 2001) implemented in the GIFT toolbox running under MATLAB. At first, each dataset was further preprocessed to remove the mean per each voxel time series and reduced by Principal Component Analysis (PCA) with the number of components set to the maximum Minimum Description Length (MDL) estimate for all datasets (Li et al., 2007). Then, a second PCA reduction was applied to all PCA components from all datasets with the number of components set to median over MDL estimates. Finally, the Infomax algorithm and ICASSO framework (Himberg et al., 2004) was used to derive maximally spatially group-specific independent components and the GICA algorithm was used to render dataset-specific spatial maps and time-series (20 runs with random initialization, minimal cluster size 16, maximal cluster size 24). We visually inspected spatial maps of the resulting components and those that represent functional networks were used for ensuing intra-NC analysis and the respective back-reconstructed time-series were used to evaluate inter-NC connectivity.

### Experimental Design and Statistical Analysis

The study was completed between September 2015 and June 2018 and was divided into three parts. In September and October, the participants underwent neuropsychological and physical assessment and fMRI data were acquired, the data acquisition took place at CEITEC, Masaryk University, and at Faculty of Sports Studies, Masaryk University in Brno.

The intervention itself took place from November to April. In May and June, the re-assessments were performed in both groups.

Two sample *t*-test and Fischer Exact tests were used to compare the demographic data and cognitive performance between the two groups before the intervention. The effect of the intervention on cognitive performance was tested using ANOVA with three factors (“subject,” “group”—DI or LAU, and “visit”—before and after intervention), with age, sex and education as covariates.

#### Intra-Network Connectivity Analysis

For intra-network connectivity analysis, the groups at baseline were first compared using ANOVA, with a group (DI or LAU) as a factor. Then, the group differences between “before” and “after” intervention were compared using ANOVA, with three factors: group (DI or LAU), subject and visit (before, after), and an interaction group x visit. Both models included three nuisance covariates (age, gender and years of education). Both models were analyzed for each selected component and the analysis included only voxels significantly active within the given network. Altogether, 20% of voxels with the highest absolute value were included in the analysis. The significance level was set to *p* < 0.05 FWE corrected (cluster level, initial cut *p* = 0.001 uncorrected) corrected for multiple comparisons using Random Field Theory (Nichols and Hayasaka, 2003).

#### Inter-Network Connectivity Analysis

For inter-network connectivity analysis, subject-specific static functional connectivity between all pairs of selected ICA components (correlation coefficient) was calculated based on the components’ time series. The resulting correlation coefficients were transformed into Z-scores using Fisher Z-transform. Again, the groups at baseline were first compared using ANOVA with a single factor—group (DI or LAU). The group differences between “before” and “after” intervention were compared using ANOVA, with three factors: group (DI or LAU), subject and visit (before, after) and an interaction group x visit. The main effect of all three factors and interaction of group (DI or LAU) and “visit” (before, after) was modeled. For both models, three covariates were included (age, gender and years of education). Both models were calculated for each connection (altogether 36 connections, 9 components between each other). The significance level was set to *p* < 0.05 with Bonferroni corrections on two contrasts (*p* < 0.025, 2 × main effect of a factor “group” (DI or LAU) or 2 × interaction “group” X “visit”).

All ANOVAs with three factors included the “subject” factor, which modeled an effect of repeated measures. In case of any significant group vs. visit results, we also performed a correlation between changes in the rs-FC and cognitive domain z-scores in the DI group. The connectivity changes were corrected for age, sex and education.

## Results

### Participant Characteristics

Altogether, 147 subjects were recruited, 120 were included in the study after reviewing the inclusion and exclusion criteria. Eleven participants included in the DI group did not meet the criterion of the minimum attendance (66.6%), those whose attendance was lower than 10% were offered to be moved to the LAU group (6 participants). Eleven participants of the LAU group were lost to follow-up.

In the present study, we analyzed data from 68 subjects from the final cohort who had good quality fMRI data, 36 DI participants and 32 LAU participants. There were no differences in age, education and MOCA scores (two sample *t*-test, *p* < 0.05) and men/women ratio (Fischer Exact test, chi square = 1.24, *p* = 0.265) between the two groups. A significant difference between the groups was found for gender distribution (Fischer Exact test, chi square = 3.95, *p* = 0.047). Both DI and LAU were heterogeneous groups consisting of both healthy controls (HC) and subjects with mild cognitive impairment (MCI). There was no difference between the DI and LAU groups in healthy seniors (HS) and MCI ratio.

### Cognitive Outcomes

The results of cognitive examinations are summarized in Supplementary Table 1. There were no differences in cognitive domain Z scores between the groups before the intervention (two sample *t*-test, *p* < 0.05) (see Table 1).

**TABLE 1:** Baseline demographic and cognitive examination data.

	DI (*N* = 36)	LAU (*N* = 32)	*p*-value
Age (years)	69.2/5.47	69.0/6.08	0.878
Gender (M/F)	5/31	11/21	0.047
Control/MCI	27/9	20/12	0.265
Education (years)	14.8/2.31	15.0/3.02	0.697
MOCA	27.2/2.81	25.9/2.93	0.069
Memory (Z score)	1.12/1.03	1.12/0.84	0.997
Attention (Z score)	0.11/0.57	0.05/0.76	0.738
Executive (Z score)	–0.36/0.64	–0.32/0.65	0.795
Visuospatial (Z score)	0.30/0.56	0.40/0.53	0.727
Language (Z score)	0.39/0.47	0.40/0.45	0.938

The interaction of factors “group” and “visit” was significant for the attention domain, with Z scores increasing in the DI group and decreasing in the LAU group (*F* = 6.00, *p* = 0.017) (see Supplementary Figure 2).

### Independent Componential Analysis Resting-State fMRI Results

Altogether 20 components were identified using the Independent Component Analysis. We visually inspected the spatial maps of the components and for further analysis of inter and intra-network connectivity, we selected those representing functional networks—cerebellum, DMN, visual network, right and left frontoparietal network, language network, salience (insulo-opercular) network, frontoparietal control network and sensory-motor network (see Supplementary Figure 1).

### Dance Intervention-Induced Changes in Intra-Network Connectivity

There were no significant differences in intra-network connectivities between DI and LAU groups at baseline. We observed significant interaction “group” x “visit” for the DMN. Based on extracted average values, the connectivity increases in the DI group and decreases in the LAU group (ANOVA, *p* = 0.048 FWE). The rs-FC in the precuneus (MNI coordinate –9, –58, 25) increased in the DI group and it decreased in the LAU group (*p* = 0.048) (see Supplementary Figure 3).

### Dance Intervention-Induced Changes in Inter-Network Connectivity

There were three significant differences in inter-NC between the DI and LAU groups before the intervention, in all three the connectivity was higher in the LAU group compared to the DI group (cerebellum –visual network—*p* = 0.0088, cerebellum—language—*p* = 0.01, language—sensory-motor network—*p* = 0.0218).

**FIGURE 1:**
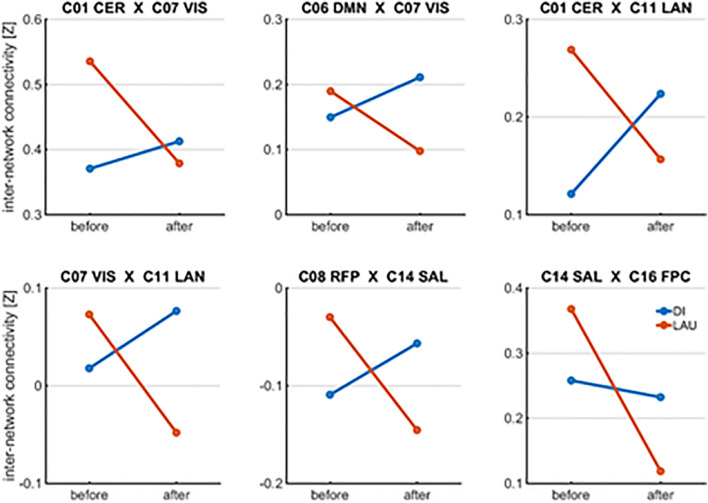
Significant results for group vs. visit interaction in inter-NC.

We found six significant results for the group vs. visit interaction. Based on the extracted average values, the inter-NC increased in the DI group (five results) and decreased in the LAU group (one result). The inter-NC between the networks decreased significantly more in LAU than in the DI group (see Table 2 and Figure 1). Briefly, DI, when compared to LAU, induced increases in the crosstalk between the insulo-opercular and right frontoparietal/frontoparietal control networks (*p* = 0.019 and *p* = 0.023), visual and language/DMN networks (*p* = 0.012 and *p* = 0.015), and cerebellar and visual/language networks (*p* = 0.015 and *p* = 0.003).

**TABLE 2:** The significant results in inter-NC connectivity between the pairs of networks.

Networks	*P*-value
Cerebellum vs. visual network	0.0147
Default mode network vs. visual network	0.0149
Cerebellum vs. language network	0.003
Visual vs. language network	0.0119
Right frontoparietal vs. salience network	0.0186
Salience network vs. frontoparietal control network	0.023

### Association Between Resting-State Functional Connectivity Changes and Cognitive Outcomes of Interest

**FIGURE 2:**
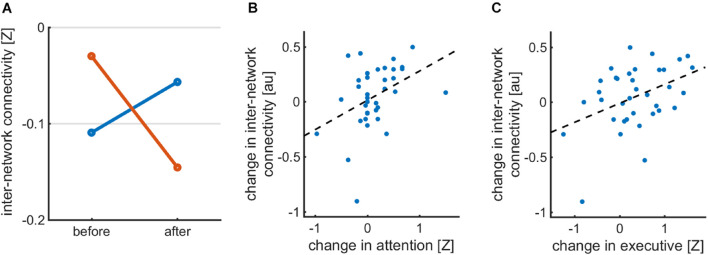
**(A)** Inter-network connectivity between RFPN and SAL networks in DI and LAU groups before and after the intervention **(B)** correlation between inter-NC between FRP and SAL in DI group and z-scores in attention domain **(C)** correlation between inter-NC between FRP and SAL in DI group and z-scores in executive domain.

The DI-induced changes in inter-NC between the right frontoparietal network and salience network significantly correlated with changes in attention domain z-scores (*R* = 0.402, *p* = 0.015) and executive function domains (*R* = 0.412, *p* = 0.012) in the DI group (see Figures 2A–C).

## Discussion

The significant behavioral effect of DI was found in the attention domain, which is in concordance with previous research (Diamond, 2015; Xiong et al., 2021) and our other research, which was performed in the same final cohort with a larger sample size (Kropacova et al., 2019). The current cohort remains smaller due to discarding data from participants with motion artifacts during fMRI scanning.

Our main goal was to assess brain rsFC changes that reflect DI-induced ability to enhance the recruitment of relevant cognitive large-scale brain networks and/or communication between them. For this purpose, we analyzed rsFC changes within specific networks as well as those of individual network pairings. We observed group vs. visit interaction for the DMN intra-network connectivity which increased in the DI group and decreased in the LAU group. DMN is a main resting state cognitive network with high degrees of functional connectivity (Greicius et al., 2003). Reduced rsFC within DMN has been described in both normal and pathological aging (such as in MCI or Alzheimer’s disease), with DMN having a strong correlation with cognitive processes (Agosta et al., 2012; Krajcovicova et al., 2014; Vidal-PiÃ±eiro et al., 2014; La et al., 2015; Qi et al., 2018a; Zonneveld et al., 2019). The positive effect of exercise, both short and long-term, on the plasticity of DMN, has already been described by several studies (Boraxbekk et al., 2016; Li et al., 2017; McGregor et al., 2018).

We found more significant results for the DI-induced rs-FC enhancement of the inter-network crosstalk than of the intra-network connectivity. This finding may relate to the fact that aging is associated with the network de-differentiation and enhanced inter-network pairing, which is one of the well-described neural compensatory mechanisms for decreased modularity and network segregation with age (Geerligs et al., 2015). While several significant results have been found for the group vs. visit interaction, only the inter-network rsFC increases between right frontoparietal network and salience (insulo-opecular) network in the DI group were associated with our cognitive outcome measures, i.e., with z-scores in attention and executive function domains. The salience network comprises anterior cingulate and ventral anterior insular cortices, bilateral Rolandic opercula, with nodes in the amygdala, thalamus, hypothalamus, ventral striatum and specific brainstem nuclei (Menon, 2015; Blefari et al., 2017; Seeley, 2019). The right FP network is located within the right frontal and parietal cortices. Both salience/fronto-opercular and right FP networks (the latter is also referred to as the ventral attention network (Farrant and Uddin, 2015) are engaged in the control of salient (i.e., behaviorally relevant) stimuli processing from internal (interoceptive awareness and bodily self-consciousness) as well as external (outside world) environment (Blefari et al., 2017; Seeley, 2019). They also play important roles in the control of working memory (Wallis et al., 2015). ACC is repeatedly associated with executive functions (Carter et al., 1999), the anterior insula is also functionally connected with frontal regions implicated in executive functions (Eckert et al., 2009). The finding that the DI enhanced this specific inter-network crosstalk related to attention to behaviorally relevant stimuli and to executive functions is novel and future studies should assess the long-term cognitive and behavioral sequelae of these changes.

We also identified increased rsFC between the cerebellum and visual/language networks, and between DMN and visual/language networks. Some of these results have to be taken with caution because of the significant differences in the inter-network connectivity strength between DI and LAU groups already at baseline. On the whole, the abovementioned networks are known to be engaged in movement coordination, and visual and speech processing (Solé et al., 2010; van den Heuvel and Hulshoff Pol, 2010; Manto et al., 2012), thus supporting the notion that DI is an enjoyable multimodal cognitive, movement and social activity that modulates brain plasticity in a specific behaviorally relevant manner.

### Study Limitations

One of the study limitations of this study is that there was no active control for the DI group, who would perform, e.g., aerobic activity, such as jogging (Müller et al., 2017). Moreover, due to the low number of MCI subjects in both DI and LAU groups, we could not perform analyses for these groups separately.

In conclusion, dance is a multimodal activity that led to complex intervention-specific brain plasticity changes that were of cognitive relevance.

## Data Availability Statement

The original contributions presented in the study are included in the article/Supplementary Material, further inquiries can be directed to the corresponding author/s.

## Ethics Statement

The studies involving human participants were reviewed and approved by the Masaryk University Ethical Committee. The patients/participants provided their written informed consent to participate in this study.

## Author Contributions

IR and ZB contributed to conception, design of the study, and wrote the first draft of the manuscript. ZB, L’N, NN-E, SK, LB, RG, PV, and LS participated in data acquisition. RG, PV, and LS were responsible for DI program preparation and evaluation. RM performed statistical analysis of data. L’N, RM, and IR wrote sections of the manuscript. All authors contributed to manuscript revision, read, and approved the submitted version.

## Conflict of Interest

The authors declare that the research was conducted in the absence of any commercial or financial relationships that could be construed as a potential conflict of interest.

## Publisher’s Note

All claims expressed in this article are solely those of the authors and do not necessarily represent those of their affiliated organizations, or those of the publisher, the editors and the reviewers. Any product that may be evaluated in this article, or claim that may be made by its manufacturer, is not guaranteed or endorsed by the publisher.
