# Supplementary material for: Dance Intervention Impact on Brain Plasticity: A Randomized 6-Month fMRI Study in Non-expert Older Adults
Source: Front Aging Neurosci. 2021 Oct 27;13:724064. doi: 10.3389/fnagi.2021.724064 (PMC8579817; doi:10.3389/fnagi.2021.724064)
Supplement: Supplementary file 1 [file Table_1.DOCX]

Supplementary material:

Table S 1 Demographic and cognitive data, including after the intervention results.

|  | | **EXAM**  (N=36) | **NON-EXAM**  (N=32) | **p-value** |
| --- | --- | --- | --- | --- |
|  | Age [years] | 69.2 / 5.47 | 69.0 / 6.08 | 0.878 |
|  | Gender (M/F) | 5/31 | 11/21 | 0.047 |
|  | Control / MCI | 27/9 | 20/12 | 0.265 |
|  | Education [years] | 14.8 / 2.31 | 15.0 / 3.02 | 0.697 |
|  | MOCA | 27.2 / 2.81 | 25.9 / 2.93 | 0.069 |
| Before | Memory [Z score] | 1.12 / 1.03 | 1.12 / 0.84 | 0.997 |
|  | Attention [Z score] | 0.11 / 0.57 | 0.05 / 0.76 | 0.738 |
|  | Executive [Z score] | -0.36 / 0.64 | -0.32 / 0.65 | 0.795 |
|  | Visuospatial [Z score] | 0.30 / 0.56 | 0.40 / 0.53 | 0.727 |
|  | Language [Z score] | 0.39 / 0.47 | 0.40 / 0.45 | 0.938 |
|  | FPT HS | 28.6 / 7.81 | 31.7 / 9.35 | 0.141 |
| After | Memory [Z score] | 1.31 / 0.78 | 1.18 / 0.98 | 0.536 |
|  | Attention [Z score] | 0.24 / 0.71 | -0.07 / 0.79 | 0.087 |
|  | Executive [Z score] | -0.01 / 0.84 | -0.22 / 0.71 | 0.280 |
|  | Visuospatial [Z score] | 0.33 / 0.58 | 0.50 / 0.58 | 0.226 |
|  | Language [Z score] | 0.46 / 0.44 | 0.40 / 0.56 | 0.614 |

Neuropsychological examination

The complex neuropsychological testing evaluated global cognitive functions (Montreal Cognitive Assessment - MOCA) and five domains: **memory** (Taylor Figure Test: Immediate Recall, Delayed Recall; Wechsler Memory Scale III: Logical memory I, Logical memory II); **attention** (Wechsler Adult Intelligence Scale III: Digit span, Symbol search); **executive functions** (Five point test, Tower of Hanoi); **visuospatial functions (**Taylor Figure Test: Copy, Judgment of Line Orientation); **language (**Mississippi Aphasia Screening Test – Receptive, Expressive and Total index), activities of daily living (Bristol Activities of Daily Living Scale); and depression (Beck Depression Inventory).

Five cognitive domains (memory, attention, executive functions, visuospatial functions, and language) were inspected for cognitive decline. All of the subjects who scored below -1.5 SD in two tests in one domain compared to normative data were categorized as MCI subjects. Moreover, we applied the MCI criteria of Albert et al. and Litvan et al., according to that all subjects have: Concern regarding a change in cognition, Impairment in one or more cognitive domains, Preservation of independence in functional abilities, Not demented, plus their structural MRI scans were inspected particularly for a hippocampal atrophy. The cognitive domain Z scores were computed as the average Z scores of the tests included in the particular domain (Aarsland et al.). The same battery was used for re-test after the intervention/no-intevention period.


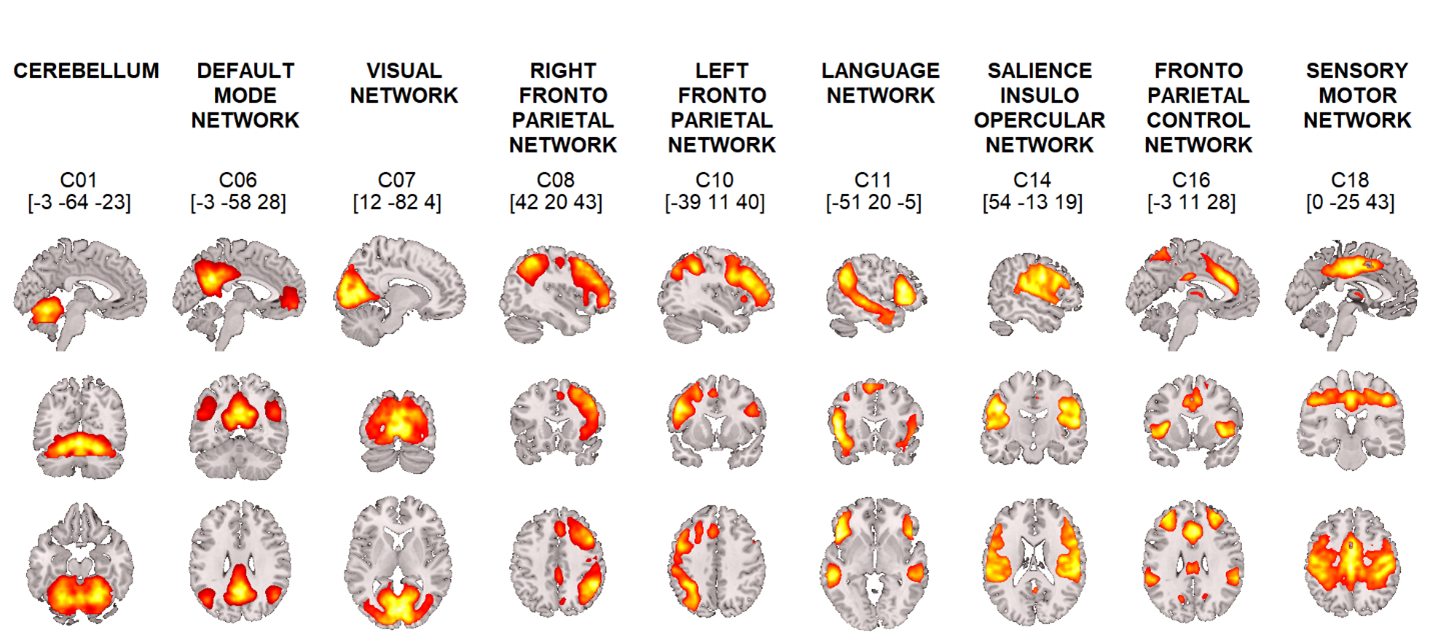


Figure S1 ICA components used for analyses.


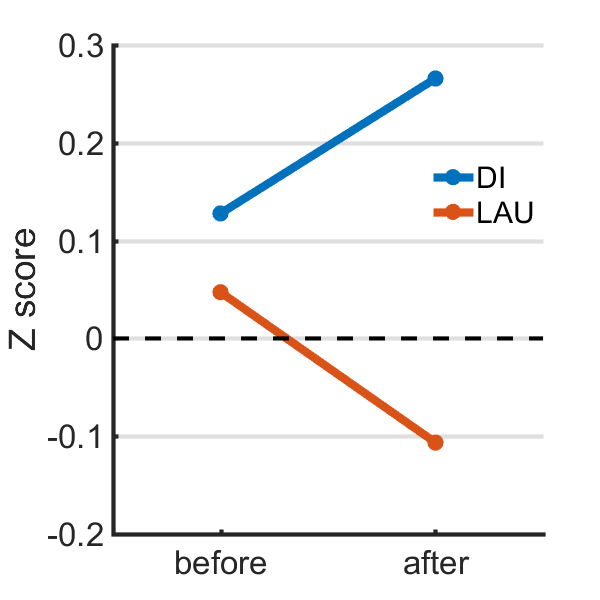


Figure S2 The effect of factor visit on z-score in attention domain in both groups


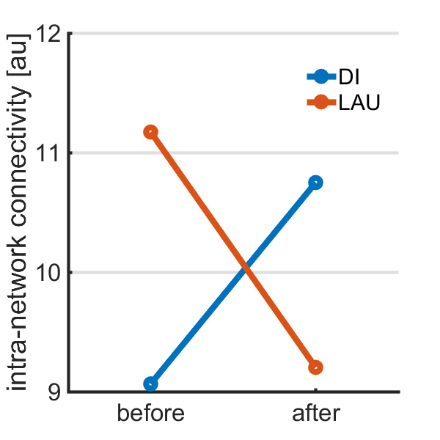


Figure S3 Interaction group v visit in DMN intra-network connectivity
